# Physical performance in older age by sex and educational level: the HUNT Study

**DOI:** 10.1186/s12877-022-03528-z

**Published:** 2022-10-26

**Authors:** Kjerstin N. Melsæter, Gro G. Tangen, Håvard K. Skjellegrind, Beatrix Vereijken, Bjørn H. Strand, Pernille Thingstad

**Affiliations:** 1 Department of Neuromedicine and Movement Science, Faculty of Medicine and Health Sciences, NTNU, Norwegian University of Science and Technology, 7491 Trondheim, Norway; 2 Trondheim Municipality, Trondheim, Norway; 3 The Norwegian National Centre for Ageing and Health, Vestfold Hospital Trust, Tønsberg, Norway; 4 Department of Geriatric Medicine, Oslo University Hospital, Oslo, Norway; 5 Department of Public Health and Nursing, Faculty of Medicine and Health Sciences, HUNT Research Centre, NTNU, Norwegian University of Science and Technology, Levanger, Norway; 6 Levanger Hospital, Nord-Trøndelag Hospital Trust, Levanger, Norway; 7 Department of Physical Health and Ageing, Norwegian Institute of Public Health, Oslo, Norway

**Keywords:** Population study, Physical function, Sex differences, Older adults, Education, Short physical performance battery, Gait speed

## Abstract

**Background:**

Population-based studies on physical performance provide important information on older people’s health but rarely include the oldest and least-healthy segment of the population. The aim of this study was to provide representative estimates of physical performance by age, sex, and educational level based on recent data from a population-based health study in Norway that includes older people with a wide range in age and function.

**Methods:**

In the fourth wave of the Trøndelag Health Study (2017–2019), all participants aged 70 + were invited to an additional examination of physical performance assessed by the Short Physical Performance Battery (SPPB), either by attending a testing station or by visits from ambulatory teams. The distribution and variation in SPPB total and subscores, as well as gait speed, are presented by sex, age, and educational level.

**Results:**

The SPPB was registered in 11,394 individuals; 54.8% were women; the age range was 70–105.4 years, with 1,891 persons aged 85 + . SPPB scores decreased by 0.27 points (men) and 0.33 points (women) for each year of age, and gait speed by 0.02 m/sec (men) and 0.03 m/sec (women). Using a frailty cut-off for gait speed at < 0.8 m/sec, the proportion of participants categorized as frail increased from 13.9% in the 70–74 years cohort to 73.9% in participants aged 85 + . Level of education $\le$ 10 years corresponded to 6 years (men) and 4 years (women) earlier onset of frailty (SPPB $\le$ 9) compared to education $\ge$ 14 years.

**Conclusion:**

We found that the SPPB captured a gradual decline and wide distribution in physical performance in old age. The results provide information about physical performance, health status, and risk profiles at a population level and can serve as reference data for clinicians, researchers, and healthcare planners.

**Supplementary Information:**

The online version contains supplementary material available at 10.1186/s12877-022-03528-z.

## Background

The current paper aims to provide updated, robust, and representative normative values for the Short Physical Performance Battery (SPPB) by age, sex, and educational level. Objective measures of physical performance, such as SPPB, are regarded as a good marker of overall health status and risk of adverse health outcomes in older people and are recommended for use in both clinical settings and research [1].

SPPB comprises three timed subtests that are each categorized to a five-point scale that also provides valid registration for those unable to perform any of the subtests [2]. The test battery is increasingly used in clinical settings and research due to its feasibility, robust measurement properties [3, 4], ability to predict adverse health outcomes in older adults, such as disability [5], mortality [4], and hospitalization [6], and for detecting and diagnosing geriatric syndromes such as frailty [7] and sarcopenia [8].

Population-based studies with assessments of SPPB are often biased towards healthier, younger, community dwelling older adults, and studies that include the full range in age and function among older people are rare. Further, improved health and function in recent cohorts of older adults imply that older normative SPPB values may not be representative for current birth cohorts [9]. Therefore, updated SPPB charts that are representative for the entire older population, including persons with frailty and persons living in institutions, are needed.

Frailty and poor physical performance are consistently reported to be more prevalent among older women than among older men [10–13]. Level of education and socioeconomic status (SES) are essential health determinants, also in older age [14–20] and individuals with less education have been found to have lower physical performance [16] and a higher likelihood of frailty compared to individuals with higher education [17]. Furthermore, earlier onset of cognitive and physical decline has been observed in older people with low and mixed SES compared to older individuals with high SES [14]. However, studies reporting how SPPB scores vary according to educational level and SES in the older population are rare and little is known about how well the SPPB reflects this health determinant.

Thus, our primary aim was to provide updated SPPB scores, both for total score and for subscores, by age, sex, and education, in a representative sample of Norway’s older population, including frail older adults at advanced age residing in nursing homes.

## Methods

### Study population

**Fig. 1:**
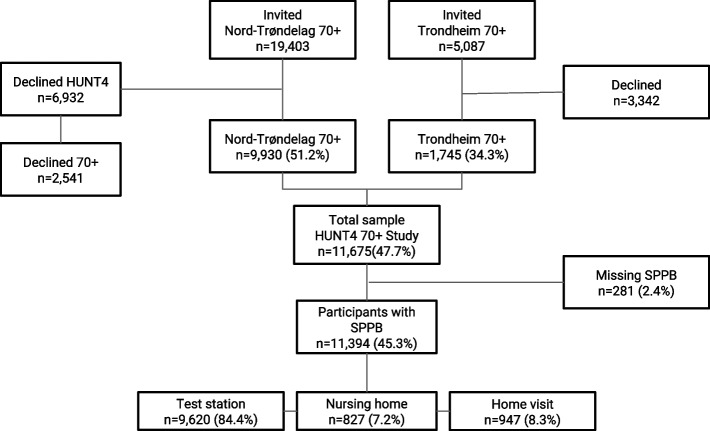
Flowchart of people invited and participating in the HUNT4 70+ study, with SPPB registrations

The study population consists of participants in the fourth wave of the Trøndelag Health Study (HUNT4) 70+ in 2017–19 (*n* = 9,930) [18] and Trondheim 70+ (*n* = 1,745). All inhabitants aged 70 + in the former Nord-Trøndelag County (23 municipalities, *n* = 19,403) and the eastern district in the city of Trondheim (*n* = 5,087) were invited to participate in these studies. Test stations were set up successively in each municipality, and for those who had problems coming to test stations, ambulatory teams provided opportunities for assessment in their own homes or nursing homes. A total of 11,675 (47.7%) participants accepted the invitation and participated. We excluded participants with no registered scores on the SPPB (*n* = 281), leaving us with 11,394 (45.3%) participants out of the 24,490 who were invited (see the flowchart in Fig. 1).

The HUNT Study is a longitudinal, population-based cohort study with data on self-reported health, health-related behavior, and demographics collected through questionnaires, clinical examinations, and interviews of the population in Mid-Norway from its start in 1984–1986 through four waves with approximately 10 years between follow-ups [18, 19]. In the fourth wave (2017–2019) of the HUNT study, all participants aged 70 years and older were invited to an additional clinical examination (HUNT4 70+) focusing on health in older age. The people in the catchment area for the HUNT study are considered representative of the Norwegian population, except for the lack of a larger city [18]. Therefore, for HUNT4 70+ , the catchment area was extended to include a district in the city of Trondheim (Trondheim 70+), which is included in the current study.

### Measures

The HUNT4 70+ and Trondheim 70+ protocols were identical and included assessments of physical and cognitive performance, in addition to a structured interview. All examinations and evaluations for each participant were conducted during the same visit. The examination took approximately 30–40 min and was performed by trained health personnel who had attended a standardized training program with practical training, use of instructional videos and written procedures. The test personnel were followed up through the data collection period to secure standardization of test procedures and quality of data.

The SPPB includes a timed hierarchic balance test, a 4-m gait speed test, and a repeated sit-to-stand test and takes approximately 5–7 min to complete [6, 20]. The balance test consists of three standing positions to be held for up to 10 s each, as follows: feet side-by-side, feet in a semi-tandem position, and feet in tandem stance. Preferred gait speed was measured twice over a 4-m distance, using the faster of the two scores, and repeated sit-to-stand was measured as the individual’s ability to rise from a chair five times at a maximal rate. If participants were unable to complete one of the assessments, they received a score of zero for the specific subtest. Based on the timing of each test, participants were given a subscore 0–4, resulting in a total score between 0 and 12, with higher scores indicating better performance. Gait speed in meters per second (m/sec) was reported as a separate variable.

Educational level was based on information from the questionnaires, where the original five categories (primary education, three years of upper-secondary school, vocational education, less than four years of college/university, more than four years of college/university) were collapsed into three groups based on the educational classification of ISCED11 and NUS 2000 [21]: primary ($\le$ 10 years of education), secondary (11–13 years of education), or tertiary ($\ge$ 14 years of education).

Information about numbers in the household, receiver of home nursing care, and self-reported health were obtained from self-reported questionnaires. Height and weight were objectively measured as part of the main HUNT4 clinical examination, and body mass index (BMI) was calculated as weight in kilos divided by height in meters squared (kg/m^2^). The Montreal Cognitive Assessment (MoCA) was performed as part of the 70 + clinical examination and included as a measure of cognitive performance.

### Statistical analysis

Data were analyzed using STATA Statistical Software version 16. Characteristics of the participants were described as mean with standard deviation (SD), median with percentiles, or frequency with percentages. Age- and sex-specific percentiles for the SPPB subscores, SPPB total score, and gait speed (m/sec) were calculated. Furthermore, for smoothing purposes, and to investigate SPPB scores by demographic characteristics, linear regression was used, and SPPB scores with accompanying 95% confidence intervals (CI) were predicted post hoc from the regression models. To allow for SPPB scores to be modeled flexibly, and to differ by age, sex, and education, we included interaction terms between sex and age and between education and age. Age was included both as a linear term and a quadratic term to allow for non-linear associations. All analyses were based on complete cases without any missing values for the included variables, varying between 91.2% and 100% of the total number of participants. Sensitivity analyses were performed to compare participants in HUNT4 who did not participate in the additional 70+ part (*n* = 2,541) and participants in HUNT4 70+ with missing values on all SPPB subtests (*n* = 281) to participants with valid SPPB score (*n* = 11,394).

### Ethics

All participants gave informed written consent to participate in the HUNT4 70+ and Trondheim 70+ . If the participants were judged to lack the capacity to give consent themselves, their closest proxy provided informed consent on the participants’ behalf. This study was approved by the Regional Committees for Medical and Health Research Ethics in Norway (REC) (id. 32333).

## Results

### Background characteristics

Table 1 describes background characteristics of participants by age and gender. SPPB was registered in 11,394 individuals, of whom 96% had valid registrations on all three subtests. Participants’ ages ranged from 70–105 years, including 1,897 participants aged 85 years or older. Most participants were evaluated at a test station (84.4%), while most of the oldest participants (89.3%) were tested in their homes or in institutions. The relative proportion of women increased over age and constituted 80% of the participants in the oldest age group. Educational levels were higher in men and higher in the younger age cohorts. Women more often lived alone and reported receiving homecare more often than men at the same age. Women reported lower self-perceived health compared to men at all ages. Women scored higher compared to men on cognitive performance in the younger age cohorts, but lower in older age cohorts.

**Table 1:**
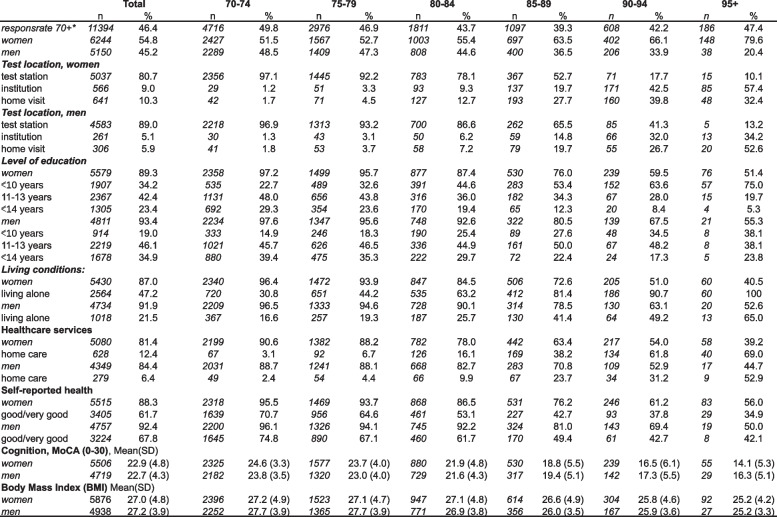
Participants characteristics by age group and sex (*N* = 11,394)

### Distribution of SPPB scores

Table 2 shows the distribution of the SPPB total score by age groups. The proportion of participants who achieved the maximum SPPB score (12 points) was 33.4%, while 4.6% were registered as unable (0 points). In the age group 70–74 years, 49.2% achieved the maximum score and 1.4% were registered as unable. In participants aged 85 years and older, 3.2% scored 12 points and 16.4% were registered as unable. None of the oldest participants (95 + years) scored higher than 9 points, and 34.6% were registered as unable.

**Table 2:**
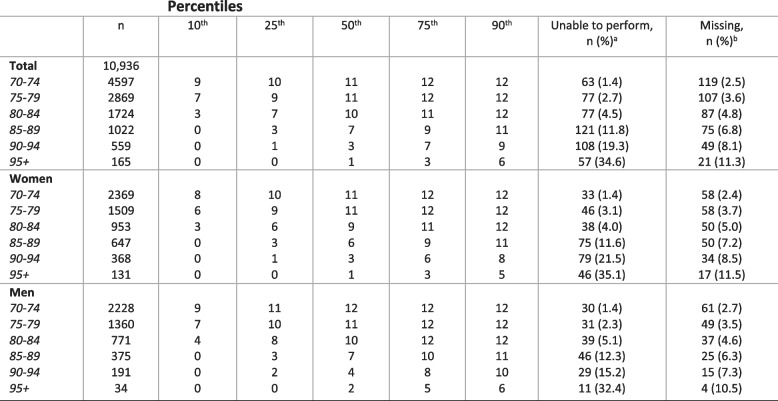
SPPB total score (0-12) percentiles based on participants with complete SPPB, by age and sex

^a^ Participants with SPPB total score 0

^b^ Participants who have scores on at least one of the SPPB subtests, but not completed the full SPPB

Tables 3, 4 and 5 shows the distribution of the timing of the three SPPB subtests. The SPPB balance subtest was the subtest with the highest proportion of participants scoring a maximum score of 4 points (66.0%). In the youngest age cohort (70–74 years), 84.0% scored 4 points and 2.6% were unable to perform the subtest (0 points). For the older participants (85 + years), 20.2% scored 4 points and 31.0% were unable. In the oldest age cohort (95 + years), 3.6% scored 4 points and 60.4% were unable to perform the subtest. The 4-m gait speed test showed a similar pattern with 81.6% scoring 4 points and 1.7% unable in the youngest age cohort (70–74 years). For participants aged 85 + years, 18.7% scored 4 points and 17.8% were unable, while among participants aged 95 + years, 1.7% scored 4 points and 33.7% were unable. The repeated sit-to-stand test was the subtest with the lowest proportion reaching the maximum score (43.3%) and was also the only subtest with a 25^th^ percentile below maximum score in the youngest age cohort (70–74 years). In participants ages 70–74 years, 59.2% scored 4 points and 4.8% were unable. In those 85 years and older, 9.8% scored 4 points and 49.8% were unable, and for participants 95 + years, none scored higher than 3 and 83.6% were unable.

**Table 3:**
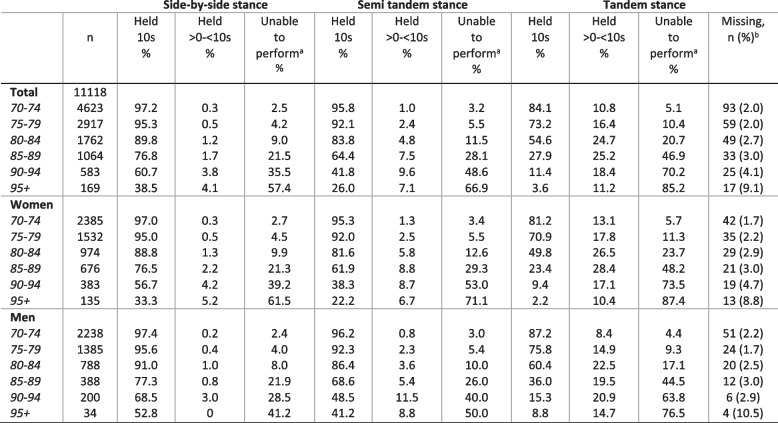
Distribution of balance tests by age and sex

Based on participants with valid registrations on the balance subtest

^a^ Participants are categorized as unable to perform if they scored 0 on the specific balance test. It includes those trying but unable to complete the test and those not able to perform the balance test at all

^b^ Participants who have score on at least one of the SPPB subtests but missing on the SPPB balance test

**Table 4:**
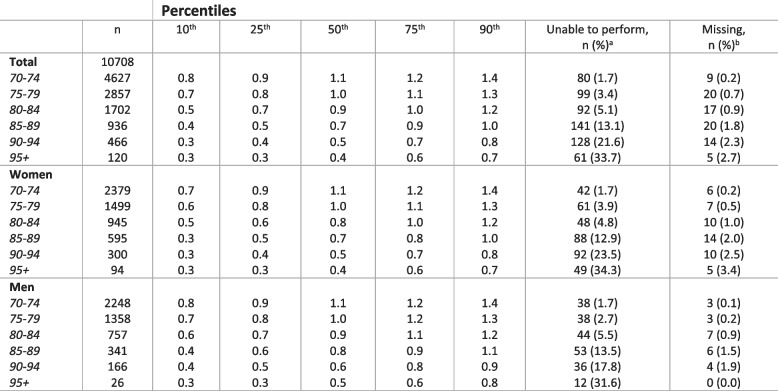
Distribution of 4-meter gait-speed test (meter/second) by age and sex

Based on participants with registered scoring on the subtest

^a^ Participants are categorized as unable to perform if they scored 0 on the SPPB gait speed test. It includes those trying but unable to complete the test and those not able to perform the gait speed test at all

^b^ Participants who have score on at least one of the SPPB subtests but missing on the gait speed test

**Table 5:**
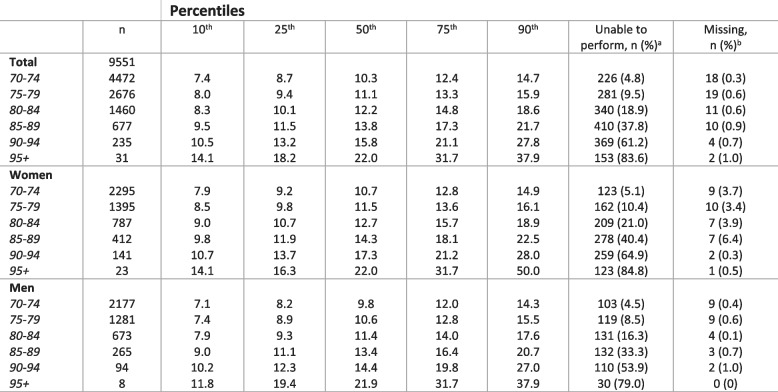
Distribution of five times repeated sit-to-stand (seconds) by age and sex

Based on participants with registered scoring on the subtest

^a^ Participants are categorized as unable to perform if they scored 0 on the SPPB sit-to-stand test. It includes those trying but unable to complete the test and those not able to perform the sit-to-stand test at all

^b^ Participants who have score on at least one of the SPPB subtests but missing on the specific sit-to-stand test

### SPPB by age, sex, and education

**Fig. 2:**
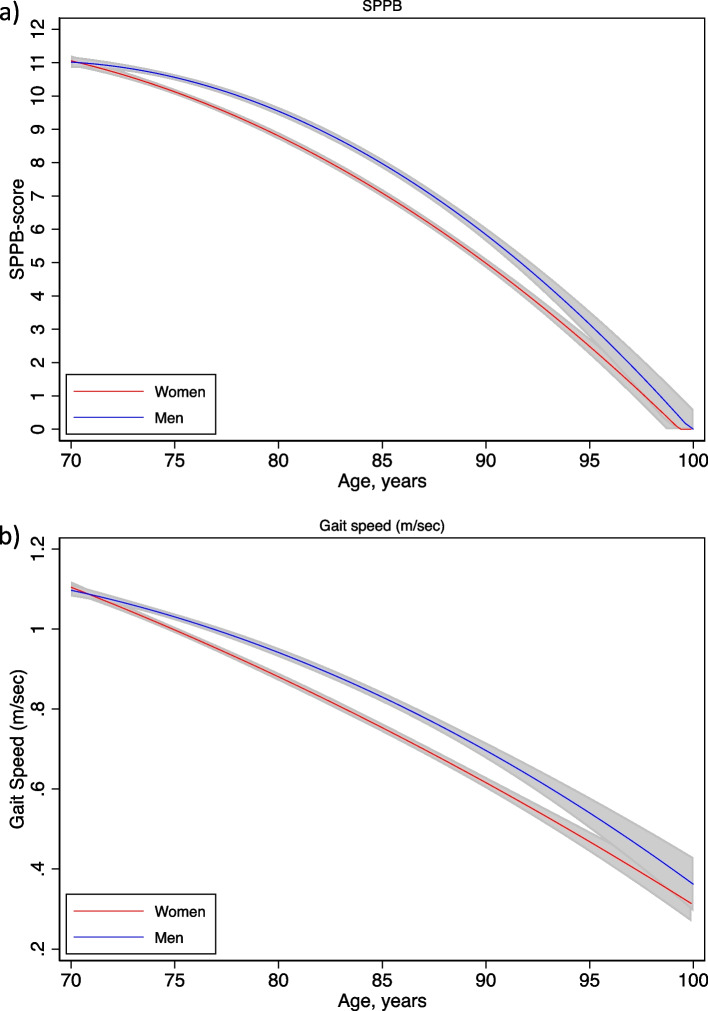
Margins plots of SPPB score (**a**) and gait speed (m/sec) (**b**) by age and sex. Predictive values with a 95% CI are shown

For every year increase in age, the SPPB score declined by 0.27 points (men) and 0.33 points (women) (Fig. 2a), and gait speed by 0.02 m/sec (men) and 0.03 m/sec (women) (Fig. 2b). Higher performance was observed for men compared to women between the ages of 72 and 96 for SPPB score and between the ages of 74 and 96 for gait speed (m/sec).

**Fig. 3:**
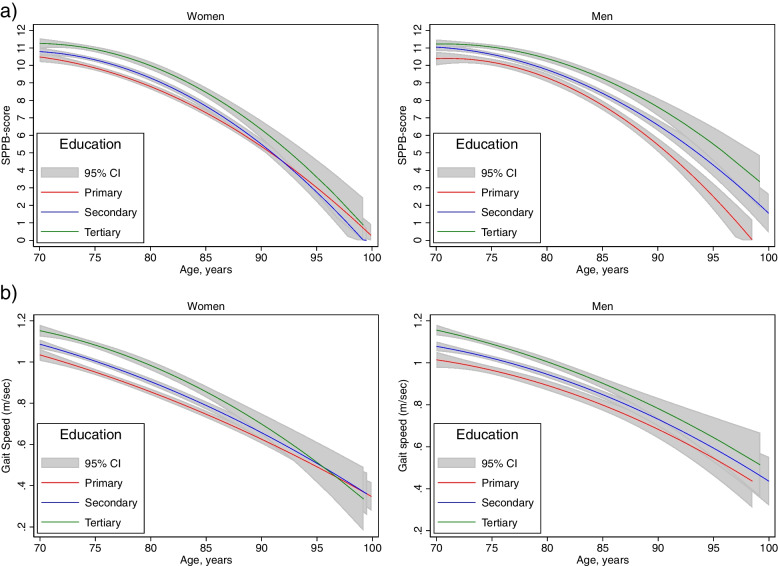
Margins plots of SPPB score (**a**) and gait speed (m/sec) (**b**) by age and level of education. Mean values with a 95% CI are shown for both men and women

The differences in the SPPB score between educational levels increased with advancing age in men and was approximately 3 points higher in men aged 85 + years with a tertiary educational level compared to those with a primary educational level. This pattern was not observed in women (Fig. 3a). For gait speed, the greatest difference between primary and tertiary educational levels was observed in the youngest participants, with a difference of 0.2 m/sec in men at ages 70–74 years. For women, the difference between tertiary and primary educational levels was highest in women at ages 80–84 years, with a difference of 0.2 m/sec in favor of women with a tertiary education (Fig. 3b). The difference between primary and tertiary educational levels corresponded to 4 and 2 years earlier onset of frailty (gait speed ≤ 0.8 m/sec) and 6 and 4 years earlier onset of frailty (SPPB score ≤ 9) in men and women respectively.

### Sensitivity analysis

Participants in HUNT4 who did not participate in the additional HUNT4 70+ part or had missing registration for the SPPB were older, 78.3 (6.3) vs 78.0 (6.5) years, the proportion with higher education was lower, 15.6% vs 25.5%, and they reported worse self-perceived health compared to those with valid SPPB scores in HUNT4 70+ .

## Discussion

This study presents updated population data (2017–2019) for the total score and subscores of the SPPB, based on a Norwegian data set of 11,394 participants aged 70–105 years. Compared to earlier published reference data for the SPPB [10], the current study includes a wider range in age and physical function, where the oldest and frailest segment of the population, as well as those living in nursing homes, are also represented.

### SPPB as a marker of age-related decline in health and function

There was a gradual decline and increased variance in total SPPB score with higher age. Most participants in the younger age cohorts scored in the upper end of the SPPB scale, with half of participants at age 70–74 reaching a maximum SPPB score. At age 85 there was a shift with a drop in the 50^th^ percentile from 10 to 7 points from the age cohorts 80–84 to 85–89 years old. For participants aged 90 + , we found that $\frac34$  scored 3 points or lower and $\frac13$ was unable to perform any of the subtests.

These observations are in support of the use of the SPPB as a marker of age-related decline in physical performance along the full range of health and function in older age. However, the observed ceiling and floor effects indicate that the SPPB is less suited for discriminating fitness level in healthy older adults or discriminate performance in older persons with severe disability. These findings are in agreement with previous studies reporting a ceiling effect of the SPPB among the youngest and healthiest participants [10, 16]. Furthermore, we observed interesting differences between the subtests with respect to proportion achieving maximum score or being unable to perform. While 66.0% of the total sample were registered with a maximum score for the balance subtest and 63.6% for the gait-speed subtest, only 43.3% scored the maximum on the repeated sit-to-stand subtest. At the lower end, the proportion unable to perform the different subtests varied from 15.7% for sit-to-stand to 4.2% for the gait speed test. In the oldest age cohort (90 + years), a relative higher proportion was unable to perform the repeated sit-to-stand subtest (70.2% women and 57.9% men) and the balance subtest (47.7% women and 41.2% men) compared to the gait-speed test (26.4% women and 20.0% men). Similar observations have been reported in other population-based studies [10, 11, 22, 23].

The possibility of scoring also those unable to perform any subtest is one of the advantages of the SPPB and part of the reason for the low missing rate. The missing rate was highest for the balance test, which is likely explained by registration routines and not by participants' characteristics. The relatively lower proportion of persons registered as unable, and amount of missing data suggest that gait speed is the subtask that captures the broadest range in function. In contrast, the sit-to-stand subtest appears to be best suited at differentiating physical performance among the healthier part of the sample.

The cut-off for scoring the maximum 4 points for the gait-speed subtest corresponds to the cut-off commonly used for defining frailty (0.8 m/sec) but is below the 1.0 m/sec cut-off indicating increased risk for adverse health outcomes [24]. Thus, there is a risk of losing information about early decline in function in the transformation from a continuous to categorical scale. This could be an argument for reporting both total and subtest scores in addition to the continuous timing variables for the gait speed and repeated sit-to-stand subtests. This aligns with a previous study suggesting that the use of gait speed as a continuous variable would allow for differentiating also among the healthier part of the older population [13].

### Effects of sex

In the present study, we found sex differences in the SPPB scores in favor of men, adding to the existing literature reporting better physical performance and less frailty in older men compared to older women, despite higher survival in women [10–12]. The observed sex differences were less marked for the youngest and oldest participants. In the younger participants this could be explained by the ceiling effect of the SPPB. In the oldest groups, there may be a more complex explanation as this group includes both individuals who have reached high age without morbidity as well as those with severe disability living in nursing homes. Overall, women demonstrated lower physical performance and a steeper decline in performance by age than men did. As a result, women were categorized as frail four years earlier than men based on SPPB score below nine [23] and six years earlier based on gait speed below 0.8 m/sec [25]. This aligns with previous reports on sex differences regarding frailty [26. ], and underscores what is known as the male–female health-survival paradox [27].

### Effects of education

Our findings of differences in physical performance depending on educational level are in line with earlier studies underscoring that education is an important health determinant also in old age [14, 16, 17]. Although Norway is characterized by well-developed healthcare and welfare systems [18], our findings indicate that these do not necessarily alleviate inequalities in health in old age. In women over 60 physical activity is related to high levels of education and income, and therefore some socio-environmental aspects of the quality of life surrounding the older person affect their physical health, but also generate inequalities in the lifestyles of older women [28].

We found the difference in physical performance in relation to educational level to be more consistent among men, which is likely related to cultural factors since higher education was less available to most women in the generation included in this study and SES in women is likely influenced by their husband’s status [29]. With older age, the difference in SPPB scores between educational groups increased and was, on average, approximately three SPPB points higher among men with tertiary education compared to primary education in participants aged 85 + years. This difference of three points indicates a substantial meaningful clinical difference with consequences for both independence in daily activities and self-perceived health [30].

### Representativeness

The participation rate for HUNT4 70+ , Nord-Trøndelag was 51.2% and for Trondheim 70+ 34.3%. Trøndelag County is found to be a good reflection of Norway based on the population composition, with general health, cause-specific mortality, unemployment rate, and educational level in this county differing little from national averages [32, 33]. Thus, the HUNT population is considered to be representative of the Norwegian population. However, Norway is not necessarily representative of other countries and geographic areas, yet it could be of special interest as socioeconomic inequalities are regarded to be low and the healthcare system is well-developed [18].

The main strengths of this study are the size of the SPPB data set, the wide range in age and function, and the low amount of missing data on the SPPB. Inclusion of a subsample from the city of Trondheim [19] has strengthened the representation of an urban population.

Recruitment is a special challenge in aging research, where barriers relate to substantial health problems, social and cultural barriers, and impaired capacity to provide informed consent which often requires cooperation from the institutions or next of kin to participate in research activities [34]. Previously, response rates in HUNT have been the lowest in the oldest and frailest inhabitants [18, 35]. In HUNT4, extra attention was paid to recruit older participants. While the overall response rate has decreased for each wave of HUNT, the response rate in the age group 80–89 years increased from 41.6 to 42.0% and in the age group 90 + from 17.2 to 43.3% from HUNT3 to HUNT4, indicating that this extra attention paid off. Sensitivity analysis showed that persons who were not included in the 70 + examination (*n* = 2,541) or had missing values on the SPPB (*n* = 281) were slightly older, had lower education, and reported slightly poorer self-reported health. Nevertheless, participants characteristics, age distribution in the sample and distribution of the SPPB scores indicate that the full range in health and function among the older population is represented.

According to national statistics, 20% of people aged 85 + are institutionalized and 66% receive home-based care in Norway [36. ]. In this study, 32% of participants aged 85 and older were tested in the institution where they lived and 32% were tested by home visits. Based on the respondents of the questionnaires, 42.2% of the 70 + sample reported they received home-based care. These observations indicate that we succeeded in including a high proportion of persons living in institutions, and among the healthier part of the older population, but could also indicate that older persons with frailty living in their own home are slightly underrepresented. This pattern is even more pronounced for the Trondheim 70+ sample where good cooperation with the staff in the institutions resulted in high participation rates for older people living in nursing homes, but lower among home dwelling older persons who may not have felt the same commitment to the HUNT study as older people in Nord-Trøndelag of whom the majority had participated in earlier waves.

The SPPB has been shown to be reliable for people with dementia [37], and there were no exclusion criteria for cognitive impairment for the present study. The testers were health personnel experienced in working with persons with cognitive impairment and the protocol and training of testers specifically addressed challenges in communication and testing of persons with cognitive impairment. The results from the testing of cognitive function (Table 2) indicate that also persons with cognitive impairment and dementia have been included. It is a strength of the study that also this part of the older population which is commonly underrepresented has been successfully included.

The amount of missing data for self-reported data on health and education are presented in Table 2 and show relatively higher amounts of missing data for the oldest age groups. This is likely due to less capacity to answer the questionnaires which were originally developed with a healthy target group in mind. This could have caused an underestimation of the effect of education in the oldest age groups but should not influence the other results.

### Clinical implications and further research

This study is a descriptive cohort study that aims to provide population data for the distribution of SPPB scores that can be utilized for comparisons and for generating hypotheses for further research. We illustrate that the SPPB is a relevant and feasible measure of physical performance that adequately distinguishes between physical performance along the continuum of decline from healthy to severely disabled in older age. Our findings add to an increasing body of knowledge suggesting that women age differently compared to men [10–12], which should be addressed in how we deliver healthcare services and plan intervention strategies. Further research on sex differences regarding responses to and effects of intervention is warranted, and our findings suggest that physical performance and SPPB can be relevant outcome measures when addressing these issues. Furthermore, our findings indicate that physical performance measured by SPPB is a relevant outcome for better understanding the impact of socioeconomic status on aging and health. In this study we used level of education as a proxy for socioeconomic status. For the oldest age cohorts for whom higher education was less available and common, and especially for women, level of education alone only partly reflects their socioeconomic status. More in-depth analysis using several sources of data, including household and children’s income is warranted. It is also of interest to explore how socioeconomic status is related to lifestyle and environmental factors like social network, living conditions and physical environment and thereby affecting e.g., physical activity behavior in old age.

## Conclusion

This study provides descriptive data for the SPPB based on a large, population-based data set that includes a wide range in age and physical function. The results add to a better understanding of the diversity in health and functioning in older age and to the knowledge about how age-related decline in physical function is affected by sex and educational level. We suggest that the SPPB is a relevant outcome measure in research on health in old age, to guide policymakers, and to develop more targeted healthcare services for older adults.

## Supplementary Information

**Additional file 1: Supplement Figure S1.** Bar graph for the different SPPB subtests.

## Data Availability

Availability and access to HUNT data from the HUNT Research Centre can be given to researchers affiliated with a Norwegian research institution if they have obtained project approval from the Regional Committee for Medical and Health Research Ethics. See https://www.ntnu.edu/hunt/data for more-detailed information on the application process and the criteria for data access.

## Abbreviations

HUNT: The Trøndelag Health Study
HUNT4: The fourth wave of the Trøndelag Health Study
SPPB: The Short Physical Performance Battery
SES: Socioeconomic status
m/sec: Meters per second
REC: Regional Committees for Medical and Health Research Ethics in Norway
BMI: Body mass index
SD: Standard deviation
CI: Confidence interval
MoCA: The Montreal Cognitive Assessment
NTNU: Norwegian University of Science and Technology
