# Supplementary figures and images for: Physical performance in older age by sex and educational level: the HUNT Study
Source: BMC Geriatr. 2022 Oct 26;22:821. doi: 10.1186/s12877-022-03528-z (PMC9597987; doi:10.1186/s12877-022-03528-z)

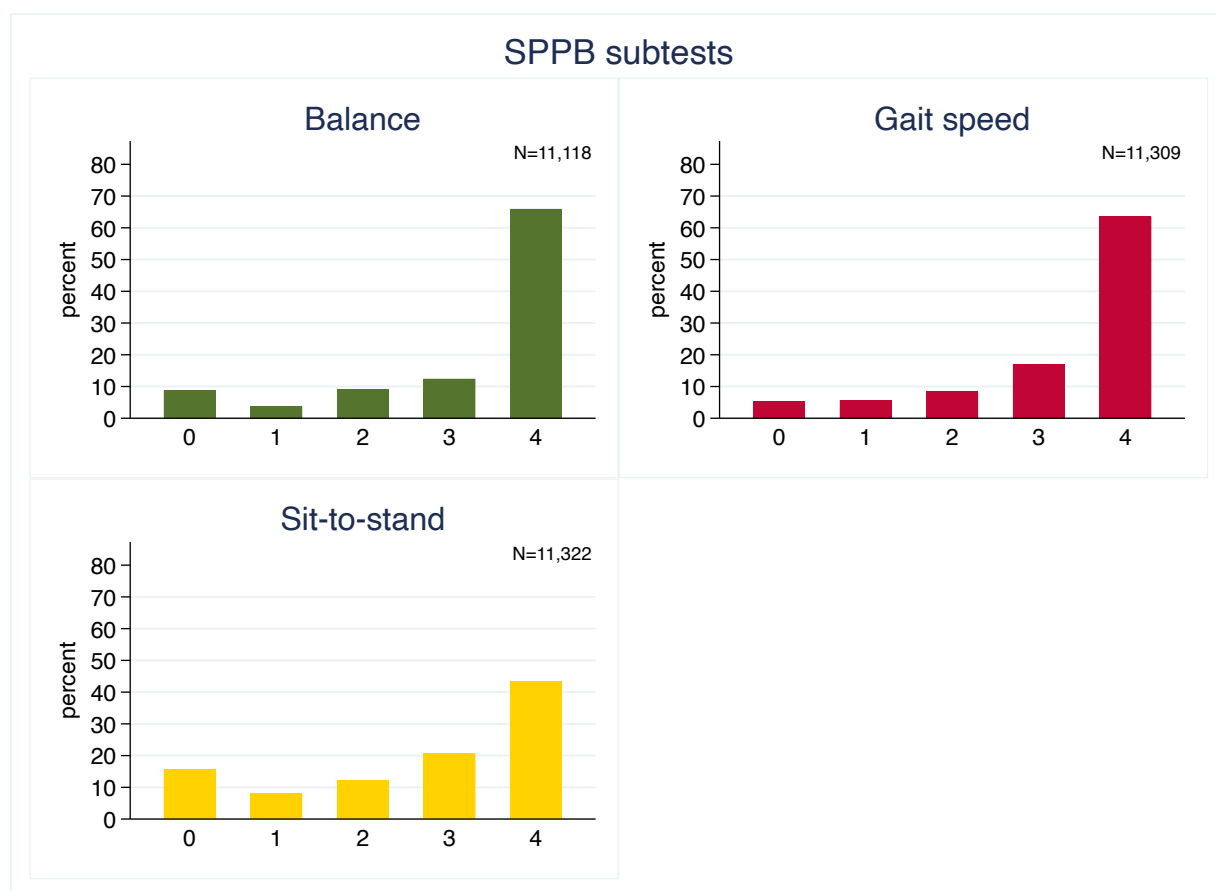

**Supplement Figure S1.** Bar graph for the different SPPB subtests.

Supplement: Supplementary file 1 — Additional file 1: Supplement Figure S1. Bar graph for the different SPPB subtests. [file 12877_2022_3528_MOESM1_ESM.pdf]
